# New species and material of Hagloidea (Insecta, Ensifera) from the Yanliao biota of China

**DOI:** 10.3897/zookeys.1033.63571

**Published:** 2021-04-22

**Authors:** Jun-Jie Gu, Xin Yang, Rong Huang, Guijun Yang, Yanli Yue, Dong Ren

**Affiliations:** 1 College of Agronomy, Sichuan Agricultural University, Chengdu, Sichuan, 611130, China Sichuan Agriculture University Chengdu China; 2 School of Life Sciences, Ningxia University,Yinchuan, 750021, China Ningxia University Yinchuan China; 3 College of Life Sciences, Capital Normal University, 105 Xisanhuanbeilu, Haidian District, Beijing, 100048, China Capital Normal University Beijing China

**Keywords:** *
Archaboilus
*, Daohugou, Cyrtophyllitinae, *
Liassophyllum
*, Middle Jurassic, Orthoptera, systematic palaeontology, Tuphellidae

## Abstract

A new species of Cyrtophyllitinae, *Archaboilus polyneurus***sp. nov.** Gu, Yue & Ren, is described from the Middle Jurassic Jiulongshan Formation, Daohugou Village, Inner Mongolia, China. The species is characterized by its ScA reaching the anterior wing margin at the level of the divergence of M+ CuA, distally branched RP, lengths of free CuA and free M equal, and numerous branches of CuA + CuPaα. A new fossil of *Liassophyllum caii* Gu & Ren, 2012 is described which increases knowledge of its wing venation and indicates that *Liassophyllum* should be assigned to the Tuphellidae.

## Introduction

The superfamily Hagloidea (Orthoptera) sensu Gorochov 1995 was widespread from the Late Triassic to the Early Cretaceous and consists of the families Haglidae, Tuphellidae, Prophalangopsidae, Hagloedischiidae (Gorochov 1995). A cladistic analysis based on wing venation suggests that it is paraphyletic (Béthoux and Nel 2002). The Prezottophlebiidae was erected by Martins-Neto (2007) and assigned to the Hagloidea on the basis of a new species from the Early Cretaceous Santana Formation of Brazil. Although Haglidae are extinct and Prophalangopsidae are now considered to be relicts, they are the most diverse Hagloidea in the fossil record (Gorochov 1995; Wappler 2001; Gu et al. 2010).

The non-marine Jurassic and Cretaceous deposits of northern China are rich and diverse in fossil insects (Wang et al. 2012; Cai and Huang 2014; Fang et al. 2020; Gao et al. 2021; Yang et al. 2021). In the Yanliao and Jehol biota, Prophalangopsidae are the most diverse and abundant Orthoptera with over thirty valid species, while the Haglidae have lower diversity and abundance. Lin (1965) described two haglid species from the Lower Jurassic of Inner Mongolia, but they were erected based on female wings, which are difficult to compare with known haglid species, which are based on males. *Alloma* Hong, 1982 (Hong 1982a) and *Hebeihagla* Hong, 1982 (Hong 1982b), are considered as synonyms of *Parahagla* Sharov, 1968 of Chifengiinae, which were originally assigned to Haglinae of Haglidae (Hong 1982a, b). The family assignment of *Yenshania hebeiensis* Hong, 1982 (Hong 1982a) is questionable, as the type specimen is very fragmentary. Although *Isfaroptera yujiagouensis* Hong, 1983 was also erected based on a very fragmentary specimen, its preserved characters are sufficient to support its assignment to Haglidae. Gu et al. (2012a, b) described two Jurassic hagloid species, *Archaboilus musicus* Gu, Engel & Ren, 2012, and *Liassophyllum caii* Gu & Ren, 2012. The broad winged species *Vitimoilus* ovatus Gu, Tian, Yin, Shi & Ren, 2017, was described from the Early Cretaceous Dabeigou Formation, the most recently described haglid species from China (Gu et al. 2017).

Here, we report a new species of the haglid subfamily Cyrtophyllitinae and describe a new fossil of *Liassophyllum caii* Gu & Ren, 2012, increasing the diversity of Haglidae and knowledge of their wing venation.

## Method and materials

The specimens were examined with a Nikon SMZ 25 microscope and photographed with a Nikon DS-Ri 2 digital camera system. Line drawings were prepared using Adobe Illustrator CC 2017 and Adobe Photoshop CC 2017 software. Measurements were taken using Adobe Illustrator. The specimens are housed at the Key Lab of Insect Evolution and Environmental Changes, Capital Normal University (CNU), Beijing, China.

Wing venation terminology follows the interpretation proposed by Béthoux and Nel (2002). Another commonly used Orthoptera venational terminology is that of Sharov (1968; and see e.g., Gorochov 1995). These mainly differ by their interpretations of the media and cubitus areas. For ease of comparison, we also provide the Sharov venation system in parentheses. Corresponding abbreviations used are: ScA (C), anterior subcosta; ScP (Sc), posterior subcosta; RA (RA), RP (Rs), anterior and posterior radius, respectively; MA (MA1), MP (MA2), anterior, posterior media, respectively; CuA (MP), CuP, anterior, posterior cubitus, respectively; CuPaα (CuA1), the anterior branch of first posterior cubitus; CuPaβ (CuA2), the posterior branch of first posterior cubitus; CuPb (CuP), the second posterior cubitus; AA1 (1A), first branch of anterior anal vein.

## Systematic palaeontology

**Class Insecta Linnaeus, 1758**

**Order Orthoptera Olivier, 1789**

**Suborder Ensifera Chopard, 1920**

**Superfamily Hagloidea Handlirsch, 1906**

### Family Haglidae Handlirsch, 1906

#### Subfamily Cyrtophyllitinae Zeuner, 1937

##### Archaboilus

Taxon classificationAnimaliaOrthopteraHagloidea

Martynov, 1937

1AFF6052-41CA-5E84-BCB1-38564F1E94CD

###### Composition.

*A. kisylkiensis* Martynov, 1937, *A. martynovi* Gorochov, 1988, *A. musicus* Gu, Engel & Ren, 2012, *A. shurabicus* Martynov, 1937, *A. similis* Zherikhin, 1985, *Archaboilus polyneurus* sp. nov.

##### Archaboilus polyneurus

Taxon classificationAnimaliaOrthopteraHagloidea

sp. nov. Gu, Yue & Ren

E46DBE62-894F-5ED9-90E8-2C4060CD0A36

http://zoobank.org/59886EC8-2ABE-4064-868D-8A0867FE5F34

Fig. 1

###### Diagnosis.

ScA reaches anterior wing margin at level of divergence of M+ CuA, RP branched distally, lengths of free CuA and free M equal, CuA + CuPaα with numerous branches.

###### Material examined.

***Holotype***, CNU-ORT-NN2009018PC. ***Paratype***, CNU-ORT-NN2009011.

###### Locality and age.

Daohugou Village, Shantou Township, Ningcheng County, Inner Mongolia, China; Jiulongshan Formation, Bathonian–Callovian boundary interval (Ren et al. 2019), Middle Jurassic.

###### Description.

**Figure 1.**
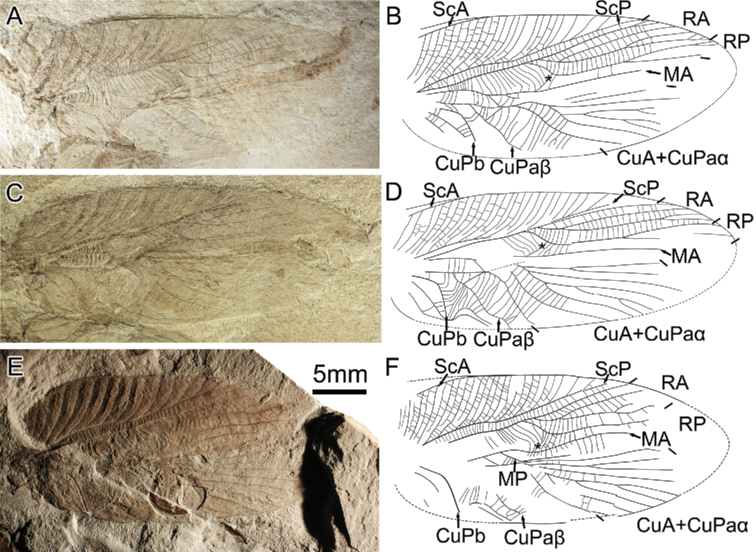
Photos and drawings of *Archaboilus polyneurus* sp. nov. Gu, Yue & Ren, asterisk indicates the transverse veinlet connecting MA and base of RP. **A–D** right and left forewing of the holotype, CNU-ORT-NN2009018C **E, F**CNU-ORT-NN2009011. Scale bar: 5 mm.

Forewing oval, estimated length ca 33 mm. ScA crossing area between ScP and anterior wing margin, reaching margin at level of divergence of M+ CuA; basal part of ScP slightly anteriorly curved, ScP reaching anterior margin at 3/4 to wing base with numerous oblique branches uniformly distributed; branches of ScP with secondary vein between them, formed by two rows of cells; most cross-veins between ScP and R straight; stem R slightly undulate; RA basally branched, pectinate with 4–7 terminal branches; base of RP curved towards to posterior margin, RP very distally branched with less branches than RA; area between RA and RP with series of regular arranged cross-veins; area between R and M expanding when R dichotomous, with series of long cross-veins, cross-veins of expanded area curved; presence of a transverse veinlet connecting MA and base of RP (asterisk on Fig. 1B, D, F); M separated from M + CuA distant to origin of RP; MA probably undulate; MP strongly curved basally (not preserved in holotype); lengths of free CuA and free M equal; CuA + CuPaα with numerous branches; CuPaβ oblique; “handle” straight; CuPb strongly oblique, basal part and middle part (where bearing teeth) forms obtuse angle.

###### Etymology.

From the Latin “*polyneurus*”, referring to its numerous branches of CuA + CuPaα.

###### Discussion.

Although the preservation and deformation of the specimens makes it difficult to identify the complete structure of ScA, this new species can be assigned to *Archaboilus* Martynov, 1937 by a combination of its ScA crossing the area between ScP and the anterior wing margin, the base of MP strongly curved, and the presence of a transverse veinlet connecting MA and the base of RP. Besides these diagnostic characters of the genus, *A. polyneurus* sp. nov. shares with *A. musicus* from the same locality a slightly sigmoidal ScP, but it differs from it by its much more distally branched RP and distinctly smaller forewing. Although the holotype and single known specimen of *A. kisylkiensis* Martynov, 1937 is only the basal half of a forewing, its free CuA is much longer than its free M, not as in the new species. *A. polyneurus* sp. nov. differs from all other *Archaboilus* species by its shorter ScA, very distally branches of RP, and numerous branches of CuA + CuPaα. Although the terminals numbers of RA and CuA + CuPaα are different between the holotype and paratype, this kind of difference has been shown to be intra-specific variation in orthopterans and their relatives (Béthoux 2008; Gu et al. 2010, 2011).

### Family Tuphellidae Gorochov, 1988

#### Genus *Liassophyllum* Zeuner, 1935

##### Liassophyllum caii

Taxon classificationAnimaliaOrthopteraHagloidea

Gu & Ren, 2012

CB5D5ACA-1D1C-5236-8CC1-EF45FD1A5598

Fig. 2

###### Material examined.

CNU-ORT-NN2020001.

###### Locality and age.

Daohugou Village, Shantou Township, Ningcheng County, Inner Mongolia, China; Jiulongshan Formation, Bathonian–Callovian boundary interval (Ren et al. 2019), Middle Jurassic.

###### Description of new material.

Isolated left forewing with negative and positive imprint; preserved length 41 mm, estimated complete length ca 49 mm, distal part of subcostal area, R, M, part of posterior margin all missing. Preserved forewing venation almost the same as previously described fossils of the species. Forewing elongated, not typically oval; the anterior wing margin is slightly flattened in its basal part, then arched upwards; there is no curved ScA crossing area between ScP and anterior wing margin; area between ScP and anterior margin basally narrowed, gradually widened to the middle; area between CuPb and CuPaβ broad, very basal cross-veins strongly curved and connected, formed into several irregular cells.

**Figure 2.**
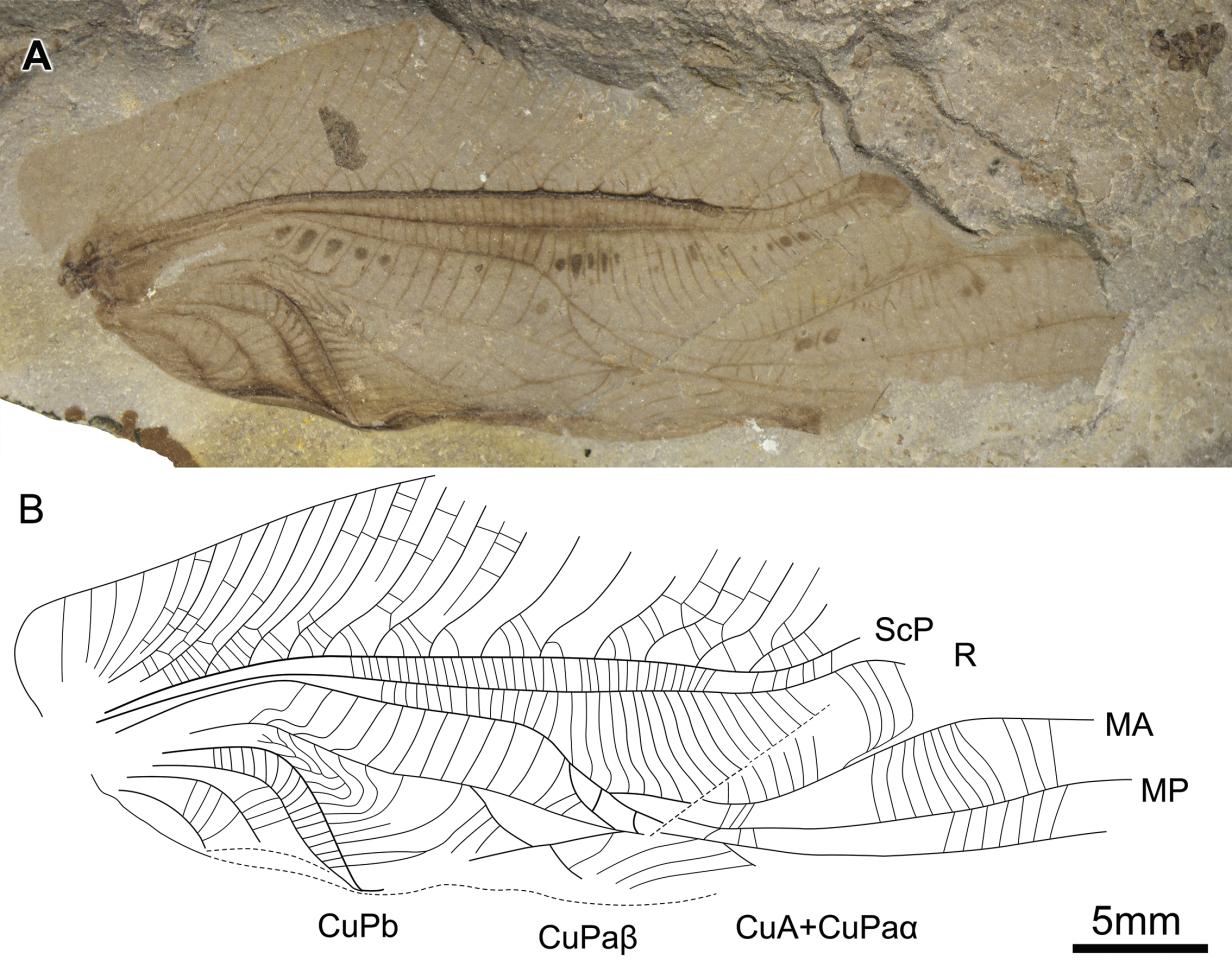
Photo (**A**) and drawing (**B**) of *Liassophyllum caii* Gu & Ren, 2012, CNU-ORT-NN2020001. Scale bar: 5 mm.

###### Discussion.

Although the distal part of the forewing is absent, we assign the new fossil to *L. caii* Gu & Ren, 2012 by the following: R is simple for a long distance and is strongly arched toward the anterior margin distal to the redirection of ScP; MA is undulate; and the area between R and MA is distinctly broad. *Liassophyllum caii* Gu & Ren, 2012 was erected based on 11 specimens. The holotype is an isolate forewing with the basal area between ScP and the anterior margin missing, the paratypes are well preserved but with wings strongly overlapped and their subcostal area is not clear. The basal part of the subcostal area of the type species *L. abbreviatum* Zeuner, 1935 is also unknown. The new material described here has a clear subcostal area, improving the knowledge of this important area. It lacks an arched ScA crossing the subcostal area positioned very close to the anterior wing margin. The basal-most area between ScP and anterior wing margin has fan-like veinlets. Zeuner (1939) and Gu et al. (2012a) attributed *Liassophyllum* to Cyrtophyllitinae, but Gorochov (1995) and Gorochov et al. (2006) excluded the genus from the subfamily, not mentioning its higher-rank assignment. The new material reported here supports exclusion of *Liassophyllum* from the Cyrtophyllitinae by its absence of an arched ScA crossing the area between ScP and the anterior margin. Further, the undulate MA, the long and more or less undulate stem of R, the very distal dichotomous R, and the broad and long area between CuPb and CuPaβ of *Liassophyllum* species strongly indicate that this genus belongs to the Tuphellidae.

## Supplementary Material

XML Treatment for Archaboilus

XML Treatment for Archaboilus polyneurus

XML Treatment for Liassophyllum caii

## References

[B1] Béthoux O Nel A (2002) Venation pattern and revision of Orthoptera sensu nov. and sister groups. Zootaxa 96: 1–88. 10.11646/zootaxa.96.1.1

[B2] Béthoux O (2008) Revision and phylogenetic affinities of the lobeattid species *bronsoni* Dana, 1864 and *silvatica* Laurentiaux & Laurentiaux-Vieira, 1980 (Pennsylvanian; Archaeorthoptera). Arthropod Systematics & Phylogeny 66: 145–163. 10.1016/j.neuron.2011.08.018

[B3] Cai CY Huang DY (2014) Diverse oxyporine rove beetles from the Early Cretaceous of China (Coleoptera: Staphylinidae). Systematic Entomology 39: 500–505. 10.1111/syen.12069

[B4] Fang H Labandeira CC Ma YM Zheng BY Ren D Wei XL Liu JX Wang YJ (2020) Lichen mimesis in mid-Mesozoic lacewings. eLife 9: e59007. 10.7554/eLife.59007 PMC7462608 32723477

[B5] Gao TP Shih CK Ren D (2021) Behaviors and interactions of insects in ecosystems of Mid-Mesozoic northeastern China. Annual Review of Entomology 66: 337–354. 10.1146/annurev-ento-072720-095043 32916066

[B6] Gorochov AV (1988) The Lower and Middle Jurassic Superfamily Hagloidea (Orthoptera). Paleontological Journal 22: 50–61.

[B7] Gorochov AV (1995) System and evolution of the suborder Ensifera (Orthoptera) (part I). Proceedings of the Zoological Institute, Russian Academy of Sciences 260: 1–224.

[B8] Gorochov AV (1996) New Mesozoic insects of the superfamily Hagloidea (Orthoptera). Paleontologicheski Zhurnal 3: 73–82.

[B9] Gorochov AV (2006) Grasshoppers and crickets (Insecta: Orthoptera) from the Lower Cretaceous of southern England. Cretaceous Research 27: 641–662. 10.1016/j.cretres.2006.03.007

[B10] Gu JJ Qiao GX Ren D (2010) Revision and new taxa of fossil Prophalangopsidae (Orthoptera: Ensifera). Journal of Orthoptera Research 19(1): 41–56. 10.1665/034.019.0110

[B11] Gu JJ Béthoux O Ren D (2011) *Longzhua loculata* n. gen. n. sp., one of the most completely documented pennsylvanian Archaeorthoptera (Insecta; Ningxia, China). Journal of Paleontology 85(2): 303–314. 10.1666/10-085.1

[B12] Gu JJ Qiao GX Ren D (2012a) The first discovery of Cyrtophyllitinae (Orthoptera, Haglidae) from the Middle Jurassic and its morphological implications. Alcheringa 36(1): 27–34. 10.1080/03115518.2011.576535

[B13] Gu JJ Montealegre ZF Robert D Engel MS Qiao GX Ren D (2012b) Wing stridulation in a Jurassic katydid (Insecta, Orthoptera) produced low-pitched musical calls to attract females. Proceedings of the National Academy of Sciences 109(10): 3868–3873. 10.1073/pnas.1118372109 PMC3309752 22315416

[B14] Gu JJ Tian H Yin XC Shi FM Ren D (2017) A new species of Cyrtophyllitinae (Insecta: Ensifera) from the Cretaceous China. Cretaceous Research 74: 151–154. 10.1016/j.cretres.2016.12.023

[B15] Hong YC (1982a) Fossil Haglidae (Orthoptera) in China. Scientia Sinica (series B) 25(10): 1118–1129.

[B16] Hong YC (1982b) Mesozoic Fossil Insets of Jiuquan Basin in Gansu Province. Geological Publishing House, Beijing, 181 pp.

[B17] Hong YC (1983) Taxonomic description. In: Rong LB (Ed.) Middle Jurassic Fossil Insects in North China.Geological Publishing House, Beijing, 42–48.

[B18] Lin QB (1965) Two insects from the Lower part of Jurassic, Inner Mongolia. Acta Palaeontologica Sinica 13(2): 363–368.

[B19] Martins-Neto RG (2007) New OrthopteraStenopelmatoidea and Hagloidea (Ensifera) from the Santana Formation (Lower Cretaceous, Northeast Brazil) with description of new taxa. Gaea 3(1): 3–8.

[B20] Martynov AB (1937) Liassic insects from Shurab and Kisyl-Kiya. Trudy Paleontologicheskovo Instituta Akademii nauk SSSR, Moscow, 7: 1–232.

[B21] Ren D Shih CK Gao TP Wang YJ Yao YZ (2019) Rhythms of Insect Evolution-Evidence from the Jurassic and Cretaceous in Northern China. Wiley Blackwell, New Jersey, 710 pp. 10.1002/9781119427957

[B22] Sharov AG (1968) Filogeniya orthopteroidnykh nasekomykh. Trudy Paleontologicheskovo Instituta Akademii nauk SSSR, Moscow, 118: 1–216.

[B23] Zherikhin VV (1985) The Jurassic Orthoptera in South Siberia and West Mongolia. In: Rasnitsyn AP (Ed.) Jurassic Insects of Siberia and Mongolia.Trudy Paleontologicheskovo Instituta Akademii nauk SSSR, Moscow, 171–184.

[B24] Wang YJ Labandeira CC Shih CK Ding QL Wang C Zhao YY Ren D (2012) Jurassic mimicry between a hangingfly and a ginkgo from China. Proceedings of the National Academy of Sciences 109(50): 20514–20519. 10.1073/pnas.1205517109 PMC3528590 23184994

[B25] Wappler T (2001) Haglidae (Insecta: Orthoptera) from the Upper Triassic Molteno Formation in southern Africa. Neues Jahrbuch für Geologie und Paläontologie – Abhandlungen 222(3): 329–352. 10.1127/njgpa/222/2001/329

[B26] Yang HR Shi CF Engel SM Zhao ZP Ren D Gao TP (2021) Early specializations for mimicry and defense in a Jurassic stick insect. National Science Review 8: nwaa056. 10.1093/nsr/nwaa056 PMC8288419 34691548

[B27] Zeuner FE (1935) The recent and fossil Prophalangopsidae. Stylops 4(5): 102–108. 10.1111/j.1365-3113.1935.tb00569.x

[B28] Zeuner FE (1939) Fossil OrthopteraEnsifera. British Museum (Natural History), London, 407 pp.

